# Characterization of the complete chloroplast genome of sunflower family species *Aster Flaccidus* (Compositae)

**DOI:** 10.1080/23802359.2019.1692710

**Published:** 2019-11-18

**Authors:** Huijuan Zhou, Ruixue She, Yuan Xu, Peng Zhao, Shuoxin Zhang

**Affiliations:** a College of Forestry, Northwest A&F University, Yangling, China;; b Key Laboratory of Resource Biology and Biotechnology in Western China, Ministry of Education, College of Life Sciences, Northwest University, Xi’an, China

**Keywords:** *Aster flaccidus*, complete chloroplast genome, Illumina sequencing

## Abstract

*Aster flaccidus* is a perennial medicinal plant belong the sunflower family Compositae, which is widely distributed in China and some other Asian countries. The complete chloroplast genome sequence of *A. flaccidus* was sequenced using the Illumina Hiseq 4000 platform. The size of the *A. flaccidus* chloroplast genome is 151,329 bp, with an average GC content of 37.5%. This circular molecule has a typical quadripartite structure containing a large single copy (LSC) region of 83,480 bp, a small single copy (SSC) region of 18,149 bp, and two inverted (IRs) repeat regions of 24,850 bp. A total of 132 genes were successfully annotated containing 87 protein-coding genes, 37 tRNA genes, 8 rRNA genes. A maximum likelihood (ML) phylogenetic tree supported that the chloroplast genome of *A. flaccidus* is closely related to that of *Aster indicus.*

*Aster flaccidus* is a perennial herb belonging to the family Compositae (Gan et al. 2006). The sunflower family (Compositae) is the largest family of complex taxonomy flowering plants, which contains more than 1600 genera and 25,000–33,000 species widely distributed in the world (Mandel et al. 2017). *Aster flaccidus* has been used as a traditional Chinese medicine for the treatment of pneumonia and pulmonary tuberculosis (Gan et al. 2006). Here we are reported first complete chloroplast genome of *A. flaccidus* based on Illumina Hiseq 4000 pair-end sequencing data.

The voucher specimen of *A. flaccidus* are stored at the herbarium of Northwest University (108°55′E, 34°15′N, accession number: SK2017179). Total genomic DNA was extracted from leaf tissue using the Plant Genomic DNA kit (Tiangen Biotech, Beijing, China). The whole-genome sequencing was conducted with 350 bp pair-end reads on the Illumina Hiseq platform (Illumina, San Diego, CA) by Novogene, Beijing, China. After trimming, the high-quality paired-end reads were assembled with MITObim v1.7 (Hahn et al. 2013) using the *A. spathulifolius* chloroplast genome sequence as a reference (GenBank number NC_027434). Annotations were performed using the online program Dual Organellar Genome Annotator (Wyman et al. 2004). The chloroplast genome sequence was deposited into GenBank (accession number MN122101).

The chloroplast genome of *A. flaccidus* was 151,329 bp in length and contains a pair of inverted repeats (IRa and IRb) regions of 24,850 bp, the large single-copy (LSC) region and small single-copy (SSC) region of 83,480 and 18,149 bp, respectively. A total of 132 genes were successfully annotated containing 87 protein-coding genes, 37 transfer RNA genes, 8 ribosomal RNA genes. Among these genes, 15 genes (*atpF*, *ndhA*, *ndhB*, *petB*, *petD*, *rpl2*, *rps12*, *rps16*, *rpoC1*, *trnI-GAU*, *trnA-UGC*, *trnK-UUU*, *trnG-UCC*, *trnL-UAA*, and *trnV-UAC*) have one intron, and two genes (*ycf3* and *clpP*) have two introns.

We constructed the maximum likelihood (ML) phylogenetic tree of the 13 Compositae complete chloroplast genome sequences obtained from NCBI using program RAxML (Stamatakis 2006). The local bootstrap probability of each branch was calculated by 1000 replications. The resulting tree showed that *A. flaccidus* was closely related to *A. indicus* with 100% bootstrap support (Figure 1).

**Figure 1.**
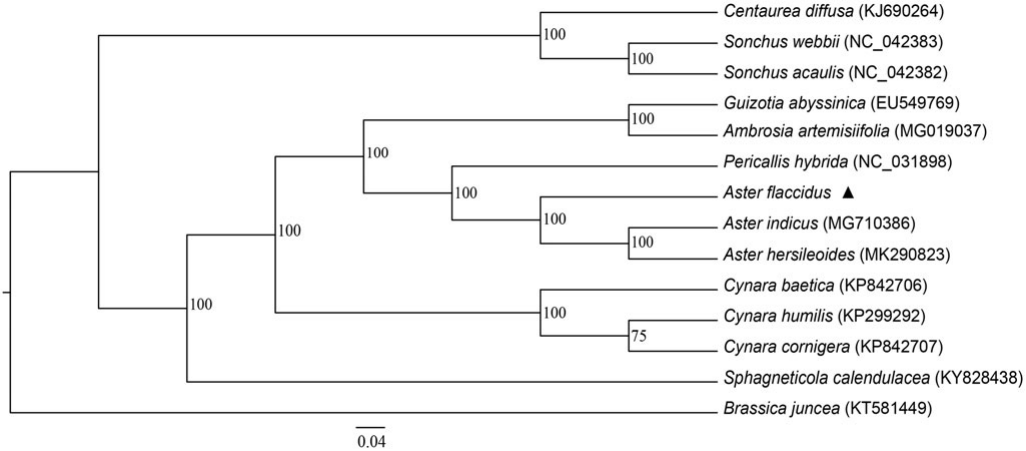
Maximum likelihood (ML) phylogenetic tree based on 14 complete chloroplast genome sequences. The accession numbers are shown in the figure, and the triangle indicates the species *A. flaccidus* used in this study.
